# The UPR inducer DPP23 inhibits the metastatic potential of MDA-MB-231 human breast cancer cells by targeting the Akt–IKK–NF-κB–MMP-9 axis

**DOI:** 10.1038/srep34134

**Published:** 2016-09-23

**Authors:** Soon Young Shin, Chang Gun Kim, You Jung Jung, Yoongho Lim, Young Han Lee

**Affiliations:** 1 Department of Biological Sciences, College of Biological Science and Biotechnology, Konkuk University, Seoul 05029, Republic of Korea; 2 Cancer and Metabolism Institute, Konkuk University, Seoul 05029, Republic of Korea; 3 Division of Bioscience and Biotechnology, College of Biological Science and Biotechnology, BMIC, Konkuk University, Seoul 05029, Republic of Korea

## Abstract

(E)-3-(3,5-dimethoxyphenyl)-1-(2-methoxyphenyl)prop-2-en-1-one (DPP23) is a synthetic polyphenol derivative that selectively induces apoptosis in cancer cells through the unfolded protein response pathway. In the present study, we evaluated the effect of DPP23 on tumour invasion and metastasis. Here, we show that DPP23 inhibited tumour necrosis factor alpha (TNFα)-induced motility, F-actin formation, and the invasive capability of MDA-MB-231 cells. DPP23 inhibited NF-κB-dependent MMP-9 expression at the transcriptional level. Akt is involved in the activation of IKK, an upstream regulator of NF-κB. DPP23 inhibited IKK and Akt, and knockdown of Akt2 significantly inhibited TNFα-induced IKK phosphorylation. We found that DPP23 bound to the catalytic domain of Akt2, as revealed by an *in silico* molecular docking analysis. These results suggest that DPP23 prevents TNFα-induced invasion of highly metastatic MDA-MB-231 breast cancer cells by inhibiting Akt–IKK–NF-κB axis-mediated MMP-9 gene expression. In addition, DPP23 attenuated experimental liver metastasis in a syngenic intrasplenic transplantation model using 4T1 mouse mammary carcinoma cells. Collectively, these results suggest that DPP23 could be used as a potential platform for the prevention of invasion and metastasis of early-stage breast cancer or as an adjuvant for chemo/radiotherapy.

Metastasis is a hallmark of malignant cancer^1^, which is characterized by the spread of primary tumour cells to distant parts of the body. Breast cancer is the most common type of female malignant disease worldwide. The highly invasive and metastatic properties of breast cancer are the main cause of death. Thus, the prevention or appropriate blockade of metastasis at early stages is important for the successful treatment of breast cancer.

Flavonoids are polyphenolic compounds that are found in many edible plants, including fruits and vegetables. Based on their chemical structures, they can be classified into several classes, such as chalcones, flavonols, flavones, flavanones, anthocyanidins, and isoflavonoids. Of these classes, chalcones (1,3,-diphenyl-2-propene-1-ones) consist of two aromatic rings, which are joined together by a three-carbon moiety^2^. Many natural or synthesized chalcones have been reported to exhibit pharmacological activities, including antitumor activity^2^,^3^,^4^,^5^,^6^,^7^,^8^. DPP23, (E)-3-(3,5-dimethoxyphenyl)-1-(2-methoxyphenyl)prop-2-en-1-one, is a synthetic chalcone derivative (Fig. 1a)^9^. We previously reported that DPP23 induces reactive oxygen species (ROS) accumulation and triggers apoptosis through the unfolded protein response (UPR) in the endoplasmic reticulum^9^. This cytotoxic effect of DPP23 has been observed in various cancer cells, including MiaPaCa-2 and Capan-1 pancreatic cancer cells, HCT116 colon cancer cells, HT1080 fibrosarcoma cells, U87MG glioma cells, and MDA-MB-231 breast cancer cells, but not in non-transformed cells. Thus, DPP23 appears to exert a general anti-tumour effect in diverse cancer cell types. However, the protective effects of DPP23 on tumour invasion and metastasis are currently unknown.

Tumour metastasis is regulated by a complex series of steps, including the disruption of the basement membrane, degradation of the surrounding extracellular matrix (ECM), increase in motility, intravasation, extravasation, and metastatic growth. The basement membrane is a fibrous, dense meshwork structure of ECM that functions to anchor the epithelium to the underlying connective tissues. Matrix metalloproteinases (MMPs) are zinc-dependent endopeptidases that are capable of degrading ECM proteins and are mainly involved in tissue remodelling. Among them, gelatinases, including MMP-2 and MMP-9, can degrade the major structural components of basement membranes, including type IV collagen^10^. The NF-κB family of transcription factors has an important role in the regulation of the transcription of many genes^11^. The NF-κB family contains five members: RelA (known as p65), RelB, c-Rel, NF-κB1, and NF-κB2. In unstimulated cells, NF-κB dimers are bound to the inhibitor of κB (IκB). Upon stimulation, the IκB kinase complex (IKK) phosphorylates IκB on Ser32, causing the rapid degradation of IκB, leading to the release of NF-κB from IκB, its translocation into the nucleus, and stimulation of its target genes. In many types of cancer, NF-κB is constitutively activated and contributes to cancer progression, including the up-regulation of MMPs^12^. The IKK complex is composed of three subunits (IKKα, IKKβ, and IKKγ). Akt (protein kinase B) is a Ser/Thr kinase downstream of phosphatidylinositol 3-kinase (PI3K), which functions as a regulator of cell proliferation and apoptosis^13^ and is involved in tumourigenesis in many cancer cells^14^. It has been reported that Akt controls NF-κB activation by activating IKK^15^,^16^,^17^,^18^,^19^,^20^.

In this study, we investigated the effects of DPP23 on anti-metastatic activity using the highly metastatic MDA-MB-231 human breast cancer cells and a syngenic experimental liver metastasis model *in vivo*. We found that DPP23 inhibits the TNFα-induced invasion of MDA-MB-231 cells by targeting the Akt-IKK-NF-κB axis to inhibit expression of the MMP-9 gene.

## Results and Discussion

### DPP23 reduces TNFα-induced migration of MDA-MB-231 breast cancer cells

**Figure 1:**
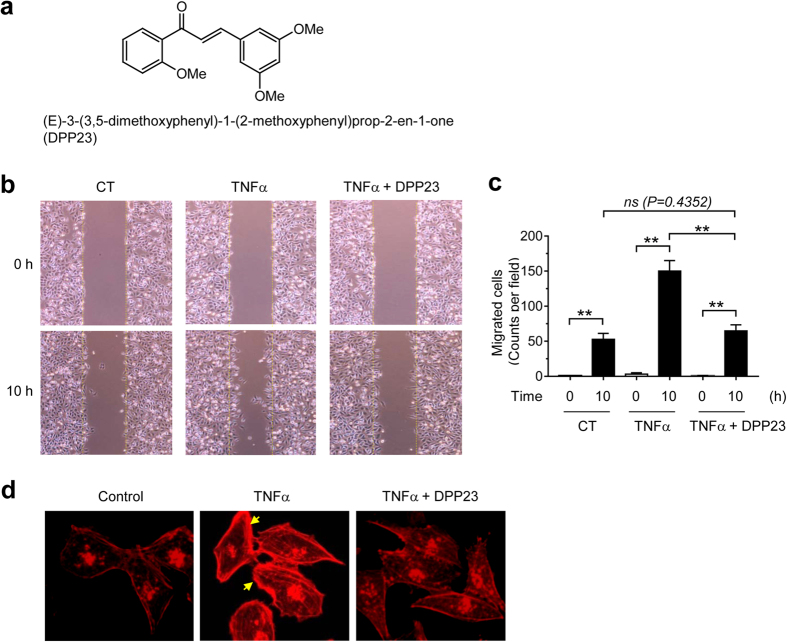
Effect of DPP23 on TNFα-induced migration of MDA-MB-231 cells. (**a**) Chemical structure of DPP23. (**b**) MDA-MB-231 cells were untreated control (CT) or treated with 10 μM DPP23 for 30 min, followed by exposure to 10 ng/mL TNFα. After 10 h, the representative field images were captured by an EVOS FL Auto Cell Imaging System. The dotted lines indicate the scraped boundaries at the beginning of the experiment. (**c**) The number of cells migrating into the scratched area was counted. Error bars represent the mean ± SD of three independent experiments. Statistical significant differences were determined by one-way ANOVA followed by Sidak’s multiple comparisons test. ***P < 0.0001 (n = 3). *ns*, not significant. (**d**) MDA-MB-231 cells were untreated or treated with 10 μM DPP23 for 30 min, followed by exposure to 10 ng/mL TNFα. After 12 h, the cells were incubated with rhodamine-phalloidin (1:100) for 1 h. The images were captured with an EVOS FL Auto Cell Imaging System. The arrows indicate polarized F-actin.

Motility is an essential feature of metastatic cancer cells^21^. In the tumour microenvironment, TNFα plays a critical role in the migration and invasion of tumour cells^22^. An *in vitro* scratch wound-healing assay was performed to determine whether DPP23 affects the TNFα-induced migration of MDA-MB-231 cells. The MDA-MB-231 monolayer was scratched with a pipette tip, and the cells were then allowed to migrate in the presence and absence of TNFα and DPP23. As expected, TNFα enhanced the migration of MDA-MB-231 cells into the scratched area compared with the migration of the untreated cells (Fig. 1b). However, TNFα-induced cell migration was significantly reduced in the presence of DPP23 (*P* < 0.0001 by one-way ANOVA followed by Sidak’s multiple comparisons test, n = 3; Fig. 1c). The actin cytoskeleton plays a key role in cell motility^23^. During cell movement, globular actin monomers (G-actin) are organized into filamentous bundles (F-actin). To determine whether DPP23 alters actin reorganization, the cells were treated with TNFα in the presence of DPP23. Polymerized F-actin was stained with rhodamine-labelled phalloidin, which selectively binds to F-actin. The cells treated with DPP23 exhibited a substantial reduction in TNFα-induced F-actin formation, mostly at the cell edges (Fig. 1d).

### DPP23 inhibits TNFα-induced invasion of MDA-MB-231-derived spheroids

**Figure 2:**
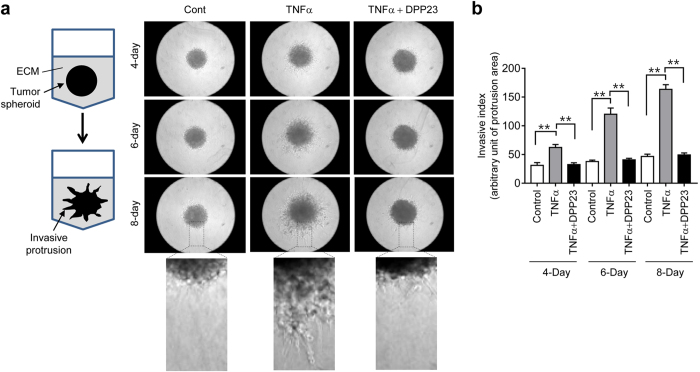
Effect of DPP23 on the invasion of MDA-MB-231 cells. (**a**) Schematic representation of three-dimensional spheroid formation and protrusion of MDA-MB-231 cells (*left*). MDA-MB-231 cell spheroids in the ECM were untreated (control) or treated with 10 ng/mL TNFα for 8 days in the presence or absence of 10 μM DPP23. The morphology of the invasive protrusions was captured with an EVOS FL Auto Cell Imaging System (*right panels*). The boxed areas are enlarged. (**b**) The invasive area was quantified using ImageJ software. Error bars represent the mean ± SD of three independent experiments. Statistical significant differences were determined by one-way ANOVA followed by Sidak’s multiple comparisons test. **P < 0.001 (n = 3).

The local invasive capability of tumour cells is considered to be an early step in the complex process of metastasis. A three-dimensional (3-D) spheroid culture system was used to determine the effect of DPP23 on tumour invasion. As shown in Fig. 2a, spheroids of MDA-MB-231 cells were not invasive under unstimulated conditions (Control). When the cells were treated with TNFα, they began to spread out of the spheroid into the surrounding matrix in a characteristic starburst pattern. However, these invasive protrusions were reduced in the presence of DPP23 (Fig. 2a). We calculated the invasive area using ImageJ software and showed that DPP23 significantly inhibited the invasive capability of MDA-MB-231 spheroids (all *P* < 0.0001 by one-way ANOVA followed by Sidak’s multiple comparisons test) (Fig. 2b).

### DPP23 downregulates TNFα-induced MMP-9 mRNA expression

**Figure 3:**
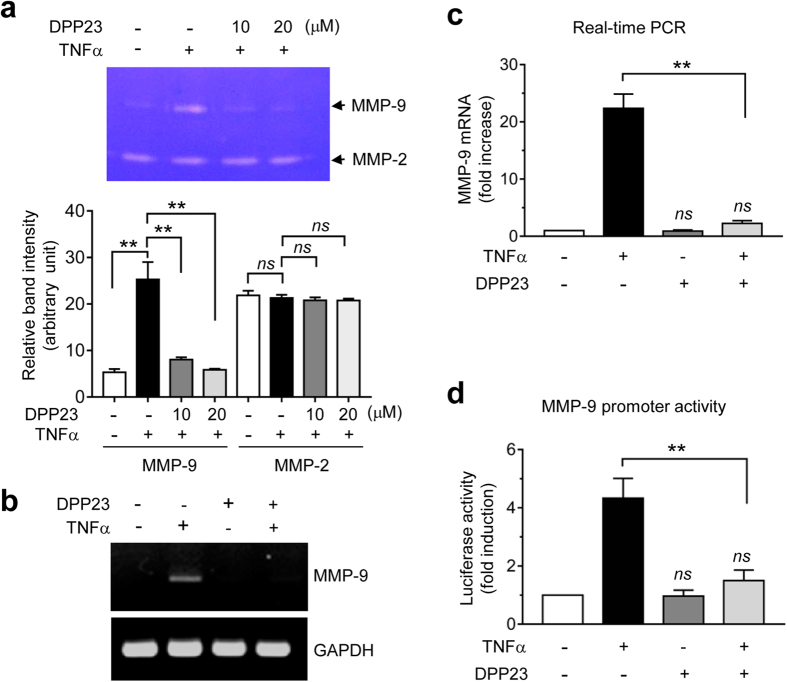
Inhibitory effect of DPP23 on TNFα-induced MMP-9 expression. (**a**) Serum-starved MDA-MB-231 cells were treated with 10 ng/mL of TNFα for 24 h in the presence or absence of DPP23 (10 or 20 μM). Conditioned media were collected, and gelatinolytic activity was measured using a 10% Zymogram gel (10% Tris-glycine/0.1% gelatin). Zymography band intensities were measured by ImageJ software and expressed as arbitrary units; background intensity level was set to “1”. The data are plotted as the mean ± SD. *P* values were determined by one-way ANOVA followed by Sidak’s multiple comparisons test (n = 3). (**b**) MDA-MB-231 cells were untreated or treated with 10 ng/mL TNFα in the presence or absence of 10 μM DPP23 for 18 h. Total RNA was isolated, and RT-PCR was then performed. The GAPDH mRNA was used as an internal control. (**c**) MDA-MB-231 cells were treated as in (**b**), and the MMP-9 mRNA levels were measured by quantitative real-time PCR. The relative fold changes were normalized to the level of the GAPDH mRNA in the same sample. The data represent the mean ± SD of three independent experiments. **P < 0.0001 by one-way ANOVA followed by Sidak’s multiple comparisons test (n = 3). *ns*, not significant versus vehicle control. (**d**) MDA-MB-231 cells were transfected with 0.2 μg of pMMP9-Luc(−925/+13) and 50 ng of pRL-null. After 48 h, the cells were treated with 10 ng/mL TNFα in the presence or absence of 10 μM DPP23 for 8 h, and the luciferase activities were then determined. Firefly luciferase activity was normalized to *Renilla* luciferase activity. The data represent the mean ± SD of three independent experiments. **P < 0.0001 by one-way ANOVA followed by Sidak’s multiple comparisons test (n = 3). *ns*, not significant versus vehicle control.

MMPs are proteolytic enzymes that degrade ECM proteins and play an important role in tissue remodelling. The basement membrane is a fibrous ECM that is important for the structural support of epithelial cells and maintains tissue organization. Gelatinases, such as MMP-2 and -9, are members of the MMP family that are able to cleave type IV collagen, a major component of the basement membrane. Gelatinase expression is highly elevated in many malignant human tumours and is strongly correlated with cancer cell invasion and metastasis^24^. To examine whether DPP23 affects MMP-2 or -9 activity, we performed an in-gel gelatinase activity assay. Gelatin zymography showed that the gelatinase activity of MMP-9 was strongly increased upon TNFα stimulation, which was significantly suppressed in the presence of DPP23 (all *P* < 0.0001 by one-way ANOVA followed by Sidak’s multiple comparisons test, n = 3; Fig. 3a). In contrast, no effect on MMP-2 gelatinase activity was observed following TNFα or DPP23 treatment. TNFα upregulates the expression of the MMP-9 gene in many cancer cells^25^,^26^,^27^,^28^. We next investigated whether DPP23 alters TNFα-induced expression of the MMP-9 gene. MDA-MB-231 cells were treated with DPP23 prior to stimulation with TNFα. The reverse transcription-polymerase chain reaction (RT-PCR) analysis showed that DPP23 alone had no effect, but substantially reduced TNFα-induced MMP-9 mRNA expression (Fig. 3b). Real-time PCR analysis was performed to precisely quantify the effect of DPP23 on MMP-9 mRNA expression. As shown in Fig. 3c, the MMP-9 mRNA level was increased by 22.4 ± 2.50-fold in response to TNFα stimulation; however, in the presence of DPP23, the level of this mRNA was significantly decreased to 2.27 ± 0.473-fold compared with the vehicle control (*P* < 0.0001 by one-way ANOVA followed by Sidak’s multiple comparisons test). To understand the possible mechanism by which DPP23 reduces MMP-9 expression, MDA-MB-231 cells were transfected with an MMP-9 promoter reporter, pMMP9-Luc(−925/+13), and the effect of DPP23 on MMP-9 promoter activity was analysed. As shown in Fig. 3d, DPP23 significantly reduced TNFα-induced MMP-9 promoter activity (*P* < 0.0001 by one-way ANOVA followed by Sidak’s multiple comparisons test). These results indicate that DPP23 has the ability to inhibit TNFα-induced MMP-9 expression at the transcriptional level.

### DPP23 suppresses TNFα-induced NF-κB activation

**Figure 4:**
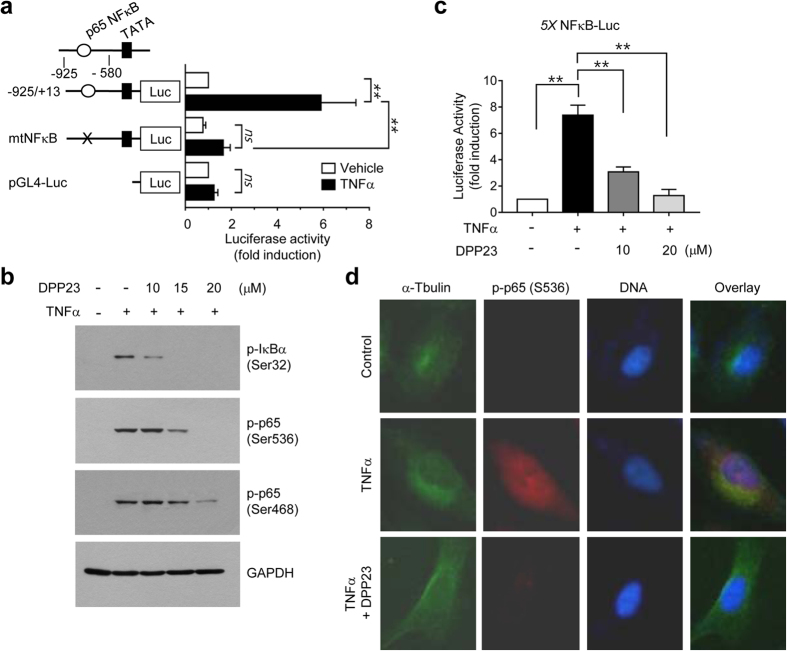
Inhibitory effect of DPP23 on TNFα-induced NF-κB activity. (**a**) MDA-MB-231 cells were transfected with 0.2 μg of pMMP9-Luc(−925/+13) and pMMP9-Luc(−925/+13)mtNFκB, along with 50 ng of pRL-null. After 48 h, the cells were treated with 10 ng/mL TNFα for 8 h, and the luciferase activities were then determined. Firefly luciferase activity was normalized to *Renilla* luciferase activity. The data represent the mean ± SD of three independent experiments performed in triplicate. **P < 0.0001 by one-way ANOVA followed by Sidak’s multiple comparisons test (n = 9). (**b**) Serum-starved MDA-MB-231 cells were pretreated with DPP23 (10, 15, or 20 μM) for 30 min before stimulation with 10 ng/mL TNFα. After 20 min, whole-cell lysates were prepared and immunoblotting was performed using phospho-specific antibodies against IκBα (Ser32), p65 (Ser536), or p65 (Ser468). GAPDH was used as an internal control. (**c**) MDA-MB-231 cells were transfected with the 5 × NFκB-Luc plasmid, along with 50 ng of pRL-null. At 48 h post-transfection, the cells were untreated or treated with 10 ng/mL TNFα in the presence or absence of DPP23 (10 or 20 μM). The data represent the mean ± SD of three independent experiments performed in triplicate. **P < 0.0001 by one-way ANOVA followed by Sidak’s multiple comparisons test (n = 9). (**d**) MDA-MB-231 cells were pretreated with 15 μM DPP23 for 30 min before stimulation with 10 ng/mL TNFα. After 20 min, the cells were fixed and incubated with antibodies against α-tubulin or phospho-p65 (Ser536) for 2 h, followed by incubation with Alexa Fluor 488-conjugated (*green signal*) or Alexa Fluor 555-conjugated (*red signal*) secondary antibodies for 30 min. Nuclear DNA was stained with 1 μg/mL Hoechst 33258 for 10 min (*blue signal*).

The MMP-9 gene is regulated by various transcription factors, including AP-1, NF-κB, SP1, Egr-1, and Ets-1^29^,^29^,^30^,^31^. Of these transcription factors, NF-κB is activated by TNFα^31^,^32^. Mutation of the NF-κB binding site within the MMP-9 gene promoter resulted in the suppression of TNFα-induced MMP-9 promoter activity (Fig. 4a), while transfection with empty vector did not affect TNFα-induced reporter activity. These data suggest that NF-κB plays a critical role in TNFα-induced MMP-9 gene transcription. To assess the possibility that the DPP23-induced suppression of MMP-9 promoter activity is associated with the inhibition of NF-κB, MDA-MB-231 cells were pretreated with DPP23 prior to the addition of TNFα, followed by the analysis of NF-κB phosphorylation. The immunoblot analysis showed that DPP23 dose-dependently reduced TNFα-induced phosphorylation of IκB on Ser32 and p65 NF-κB on both Ser536 and Ser438 (Fig. 4b), suggesting that DPP23 targets the IκB upstream kinase. We next tested whether the DPP23-induced inhibition of NF-κB phosphorylation is functionally linked to the inhibition of its transcriptional activity. MDA-MB-231 cells were transfected with an NF-κB *cis*-acting luciferase reporter containing five repeats of NF-κB binding sites (5 × NFκB-Luc). In this reporter system, luciferase activity represents NF-κB-mediated transcriptional activity. TNFα increased luciferase reporter activity 7.4 ± 0.76-fold (Fig. 4c). However, this activity was significantly reduced in the presence of DPP23 compared with the activity in the TNFα-treated group (*P* < 0.0001 by one-way ANOVA followed by Sidak’s multiple comparisons test, n = 9). To further determine the effect of DPP23 on the inhibition of NF-κB, p65 phosphorylation on Ser536 was analysed using immunofluorescence microscopy. Upon TNFα stimulation, fluorescent staining for phospho-p65 NF-κB at Ser536 was remarkably increased in the nucleus. However, when the cells were pretreated with DPP23, this phosphorylation was substantially suppressed (Fig. 4d), supporting the assumption that DPP23 inhibits MMP-9 expression by inhibiting the NF-κB-mediated pathway.

### DPP23 binds to the catalytic domain of Akt2 *in silico*

**Figure 5:**
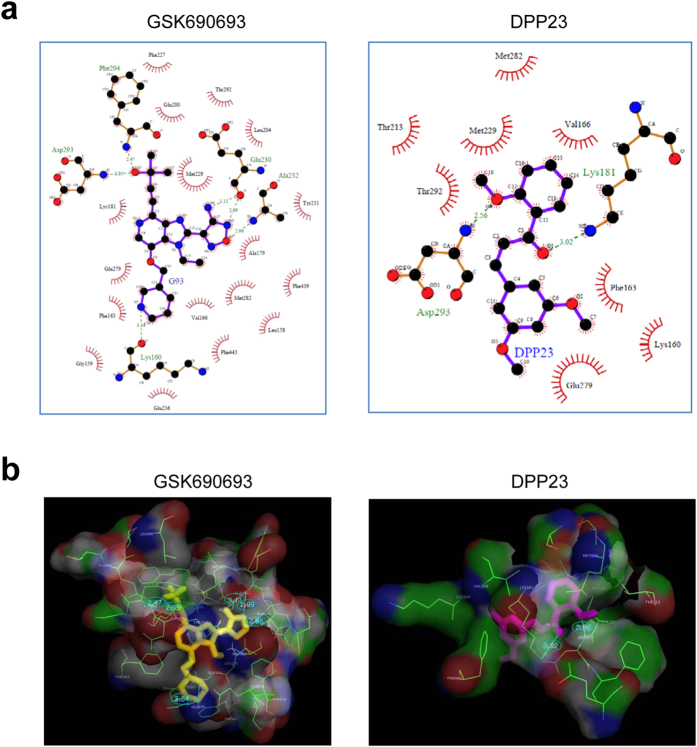
Molecular docking simulation of DPP23 binding to the catalytic domain of Akt2. (**a**) The residues of Akt2 participating in the interaction with GSK690693 (G93; *left*) and DPP23 (*right*) were analysed using the LigPlot program. The red half circles denote the residues that formed hydrophobic interactions, and the green dotted lines and numbers represent the hydrogen bonds and distances. (**b**) 3D images of the Akt2 and GSK690693 complex (*left*) and the Akt2 and DPP23 complex (*right*). GSK690693 is coloured in yellow (*left*), and DPP23 is coloured in magenta (*right*). The cyan lines represent the hydrogen bonds.

The phosphatidylinositol 3-kinase (PI3K)-Akt signalling pathway can activate NF-κB by phosphorylating IKK^15^,^16^,^17^,^18^,^19^. Three Akt isoforms (Akt1, Akt2, and Akt3) are expressed in MDA-MB-231 cells^33^. As Akt2 is the predominant form amplified in breast cancer cells^34^ and is involved in the invasion and metastasis of breast cancer cells^35^, it is tempting to speculate that DPP23 inhibits NFκB by targeting Akt2. To test this possibility, we determined whether DPP23 could bind to Akt2 using *in silico* molecular docking simulations. A pan-Akt inhibitor, GSK690693, was used as a reference ligand^36^. The LigPlot analysis showed that twenty-two residues of Akt2 (Leu158, Gly159, Lys160, Phe163, Val166, Ala179, Lys181, Glu200, Leu204, Phe227, Met229, Glu230, Tyr231, Ala232, Glu236, Glu279, Met282, Thr292, Asp293, Phe294, Phe439, and Phe443) were involved in GSK690693 binding; four residues (Lys160, Glu230, Ala232, and Asp293) formed five hydrogen bonds (H-bonds), and eighteen residues formed hydrophobic interactions (Fig. 5a, left panel). Similarly, ten residues (Lys160, Phe163, Val166, Lys181, Thr213, Met229, Glu279, Met282, Thr292, and Asp293) were located in close proximity to the DPP23 binding site (Fig. 5a, right panel). All of these residues except Thr213 were observed in both complexes, two residues (Lys181 and Asp293) formed two H-bonds, and ten residues formed hydrophobic interactions. PyMOL showed that five H-bonds were formed between Akt2 and GSK690693 (Fig. 5b, left panel), while two H-bonds were formed in the Akt2 and DPP23 complex (Fig. 5b, right panel). Based on these data, we suggest that DPP23 may bind to the catalytic domain of Akt2.

### DPP23 inhibits TNFα-induced Akt activation

**Figure 6:**
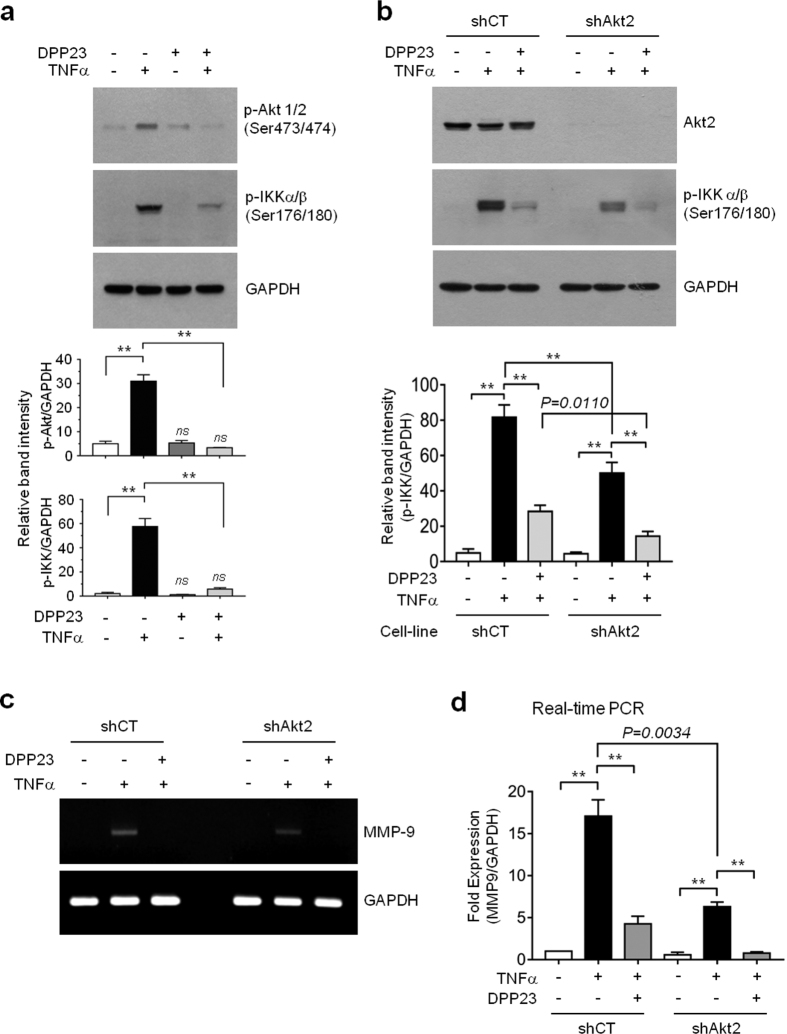
Inhibitory effect of DPP23 on TNFα-induced Akt and IKK phosphorylation and MMP-9 mRNA expression. (**a**) Serum-starved MDA-MB-231 cells were pretreated with or without 10 μM DPP23 for 30 min before stimulation with 10 ng/mL TNFα. After 20 min, whole-cell lysates were prepared and immunoblotting was performed using the phospho-specific antibodies against Akt (Ser473), p65 (Ser536), or p65 (Ser468). GAPDH was used as an internal control. Band intensities were analysed using a quantitative scanning densitometer. The data are plotted as the mean ± SD. *P* values were determined by one-way ANOVA followed by Sidak’s multiple comparisons test (n = 3). (**b**) MB231/shCT and MB231/shAkt2 cells were serum-starved for 24 h and then pretreated with 10 μM DPP23 for 30 min before stimulation with 10 ng/mL TNFα. After 20 min, the cells were lysed and immunoblotting was performed using antibodies against Akt2 and a phospho-specific antibody against IKKα/β (Ser176/180). GAPDH was used as an internal control. Band intensities were analysed using a quantitative scanning densitometer. The data are plotted as the mean ± SD. *P* values were determined by one-way ANOVA followed by Sidak’s multiple comparisons test (n = 3). (**c**,**d**) MB231/shCT and MB231/shAkt2 cells were pretreated with 10 μM DPP23 for 30 min before stimulation with 10 ng/mL TNFα. After 12 h, the cells were collected and the MMP-9 mRNA levels were measured by RT-PCR (**c**) and quantitative real-time PCR (**d**). The data are plotted as the mean ± SD. *P* values were determined by one-way ANOVA followed by Sidak’s multiple comparisons test. **P < 0.0001 (n = 9).

We next examined whether DPP23 inhibits Akt activity. For maximal activation, threonine phosphorylation in the catalytic domain (at Thr308 in Akt1 and Thr309 in Akt2, located in the activation loop) and serine phosphorylation (at Ser473 in Akt1 and Ser474 in the C-terminal hydrophobic motif of Akt) are required^37^. Figure 6a shows that TNFα increased the phosphorylation of Akt on Ser473/474 and of IKKα/β on Ser176/180 in serum-starved MDA-MB-231 cells, while treatment with DPP23 alone had no effect on their basal phosphorylation. However, when cells were pretreated with DPP23, TNFα-induced phosphorylation of Akt1/2 and IKKα/β was significantly reduced (all *P* < 0.0001 by one-way ANOVA followed by Sidak’s multiple comparisons test, n = 3). Although Akt1 is important for tumour development, Akt2 is known to play a pivotal role in tumour invasion and metastasis^38^,^39^. To further investigate the possibility that inhibition of IKK phosphorylation in response to DPP23 treatment is due to the inhibition of Akt, we established MDA-MB-231 variant cell lines that stably expressed an Akt2 shRNA (MB231/shAkt2) or scrambled shRNA (MB231/shCT). Stable knockdown of Akt2 by shRNA was evaluated by immunoblotting (Fig. 6b). Silencing of endogenous Akt2 significantly attenuated the ability of TNFα to induce IKKα/β phosphorylation (all *P* < 0.05 by one-way ANOVA followed by Sidak’s multiple comparisons test, n = 3). These findings suggest that DPP23 inhibits IKK activity by targeting Akt, resulting in the suppression of TNFα-induced NF-κB phosphorylation.

### Akt2 is involved in TNFα-induced MMP-9 transcription

RT-PCR analysis was performed on MB231/shCT and MB231/shAkt2 cells to further determine whether the suppression of TNFα-induced MMP-9 expression by DPP23 is functionally linked to the inhibition of Akt2. As shown in Fig. 6c, the induction of MMP-9 mRNA expression by TNFα was substantially reduced in MB231/shAkt2 cells compared with MB231/shCT cells. Real-time PCR analysis demonstrated a 17.07 ± 1.96-fold increase in the level of the MMP-9 mRNA in the MB231/shCT cells, whereas a 6.30 ± 0.56-fold increase was observed in MB231/shAkt2 cells following TNFα stimulation compared with the basal levels (Fig. 6d). These data suggest that Akt plays an important role in the TNFα-induced regulation of MMP-9 transcription in MDA-MB-231 cells. Thus, DPP23 reduces TNFα-induced MMP-9 expression by inhibiting the Akt–IKK–NF-κB axis in MDA-MB-231 cells.

### DPP23 suppresses experimental syngenic liver metastasis *in vivo*

**Figure 7:**
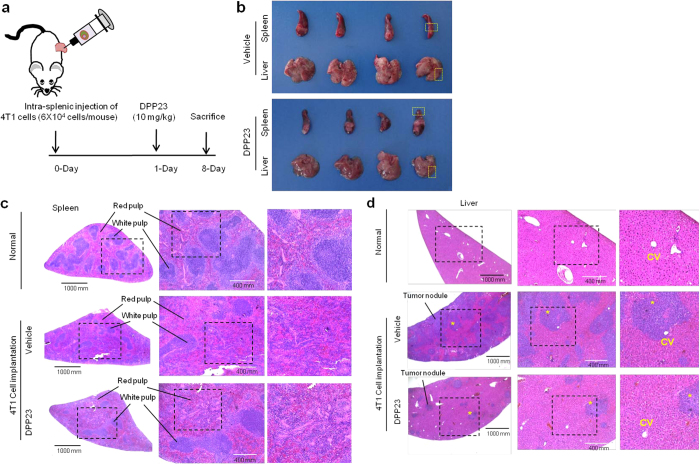
Inhibitory effect of DPP23 on syngenic liver metastasis *in vivo*. (**a**) The 4T1 mouse mammary carcinoma cells (6 × 10^4^ cells/50 μL) were injected into the splenic lobe. After 1 day, the mice were randomly divided into two groups; one group was intraperitoneally administered with PBS (n = 4), while the other group was intraperitoneally administered with 10 mg/kg DPP-23 (n = 6) daily for 7 days. (**b**) The mice were euthanized on day 8 after implantation of the 4T1 cells, and the spleen and liver tissues were fixed with formalin. (**c**,**d**) Images of histologic sections stained with H&E of spleen (**c**) and liver (**d**) from mice untreated normal (*top panels*) or 4T1-implanted mice treated with vehicle (*middle panels*) or DPP23 (*bottom panels*). CV, central vein. Scale bar, 1000 μm (left panels) or 400 μm (middle panels). *tumour nodule

We next investigated the effect of DPP23 on the anti-metastatic potential of cells using a mouse syngenic intrasplenic metastasis model. We used 4T1 mouse mammary carcinoma cells, which are known to be highly metastatic to the liver^40^,^41^. On day 1 after the intrasplenic injection of 4T1 cells, the mice were randomly divided into two groups, and phosphate-buffered saline (PBS; n = 4) or 10 mg/kg DPP23 (n = 6) was inoculated in the peritoneum daily for 7 days (Fig. 7a). All mice were euthanized on day 8 after implantation (Fig. 7b). Hematoxylin-Eosin (H&E) staining revealed the marked diffuse expansion of 4T1 cells around the red pulp region of the spleen in both vehicle- and DPP23-treated mice (Fig. 7c). Liver metastasis of 4T1 cells injected to spleen was observed based on markedly increased formation of tumour nodules in the liver of PBS-treated mice (Fig. 7d, middle panels). In contrast, smaller tumour nodules were observed in the liver of DPP23-treated mice (Fig. 7d, bottom panels). These data suggest that DPP23 has a potent inhibitory effect on the experimental liver metastasis of mouse mammary tumour cells.

In summary, this study shows that DPP23 inhibits the TNFα-induced invasion of highly metastatic MDA-MB-231 breast cancer cells by targeting the Akt–IKK–NF-κB axis to inhibit the expression of the MMP-9 gene. We also found that DPP23 attenuates experimental liver metastasis in a syngenic intrasplenic transplantation model using 4T1 mouse mammary carcinoma cells. This finding suggests that DPP23 could be used to prevent the invasion and metastasis of early-stage breast cancer or as an adjuvant for chemo/radiotherapy.

## Methods

### Cells and chemicals

Human breast carcinoma cells (MDA-MB-231) were obtained from the American Type Culture Collection (ATCC, Rockville, MD, USA). Mouse mammary carcinoma cells (4T1) were kindly provided by Dr. Jeong-Seok Nam (Gwangju Institute of Science and Technology, Gwangju, ROK). The cells were grown in Dulbecco’s modified Eagle’s medium (DMEM) supplemented with 10% (v/v) heat-inactivated foetal bovine serum (Cellgro, Manassas, VA, USA). TNFα was purchased from Sigma-Aldrich (St. Louis, MO, USA). The firefly and *Renilla* Dual-Glo^™^ Luciferase Assay System was purchased from Promega (Madison, WI, USA). The pRL-null plasmid, which encodes *Renilla* luciferase, was also purchased from Promega.

### Cell migration assay

The migration of MDA-MB-231 cells was determined using an *in vitro* scratch-wound healing assay, as previously described^42^. In brief, a scratch was made on a monolayer of cells with a micropipette tip, and the cells were untreated (control) or treated with 20 ng/mL TNFα in the presence or absence of 10 μM DPP23. After 10 h, the cells were photographed with an EVOS^®^ FL Auto Cell Imaging System (Life Technologies, Carlsbad, CA, USA). Cell migratory ability was determined by counting the number of cells invading the scratch-wound area. P value was determined by one-way ANOVA followed by Sidak’s multiple comparisons test.

### Actin reorganization

MDA-MB-231 cells grown on glass coverslips were treated with 10 ng/mL TNFα for 12 h in the presence or absence of 10 μM DPP23. F-actin was stained using a Rhodamine-Phalloidin-based F-Actin Visualization Biochem Kit^TM^ (Cytoskeleton, Inc., Denver, CO), according to the manufacturer’s instructions. Fluorescence was analysed with an EVOS^®^ FL Auto Cell Imaging System (Life Technologies).

### 3-D spheroid culture and invasion assay

The 3-D invasion assay was performed using the Cultrex 3-D Spheroid Cell Invasion Assay kit (Trevigen, Inc., Gaithersburg, MD, USA), as previously described^7^. Briefly, MDA-MB-231 cell spheroids were formed, embedded in Matrigel-based ECM, and treated with or without 10 ng/mL TNFα in the presence or absence of 10 μM DPP23. Invasive protrusions into the ECM were visualized with an EVOS^®^ FL Auto Cell Imaging System (Life Technologies), and the invasive area was quantified using ImageJ software (NIH).

### RT-PCR and quantitative real-time PCR

Total RNA was extracted using the Isol-RNA lysis reagent (NucleoZOL; Clontech, Mountain View, CA, USA), and PCR was performed as previously described^43^. For quantitative real-time PCR, the TaqMan-iQ^TM^ supermix kit (Bio-Rad, Hercules, CA, USA) was used with the Bio-Rad iCycler iQ^TM^ thermal cycler, according to the manufacturer’s instructions. The TaqMan^TM^ fluorogenic probes and gene-specific PCR primers for MMP-9 and glyceraldehyde-3-phosphate dehydrogenase (GAPDH) are described elsewhere^43^. The relative fold changes were normalized to the level of the GAPDH mRNA in the same sample.

### In-gel gelatinase assay

Gelatin zymography was performed using the Novex^®^ Zymogram gelatin gel system (Novex, San Diego, CA, USA) according to the manufacturer’s instructions. Briefly, serum-starved MDA-MB-231 cells were treated with 10 ng/mL TNFα for 24 h in the presence or absence of DPP23. The conditioned medium was collected and subjected to polyacrylamide gel electrophoresis using 10% Novex^®^ pre-cast gels (10% Tris-glycine gel/0.1% gelatin) under nonreducing conditions. After electrophoresis, the proteolytic activity of the MMP enzymes on gelatin was measured as previously reported^31^. Zymography band intensities were expressed as arbitrary units, which set “1” as a background intensity.

### NF-κB-dependent transcriptional activity assay

MDA-MB-231 cells were transfected with 0.1 μg of the 5 × NFκB-Luc plasmid, which contains five repeats of NF-κB binding sites. At 24 h post-transfection, the cells were treated with 10 ng/mL TNFα in the presence or absence of DPP23 (10 and 20 μM), as previously described^7^. The luciferase activities were measured with a Centro LB960 luminometer (Berthold Technologies; Bad Wildbad, Germany).

### Immunoblot analysis

The cells were lysed, and the proteins were separated via SDS-polyacrylamide gel electrophoresis, transferred to nitrocellulose membranes, and incubated with appropriate primary and secondary antibodies. Primary antibodies against phospho-IKKα/β (Ser176/180), phospho-IκB (Ser32), phospho-p65 (Ser536), and phospho-p65 (Ser468) were obtained from Cell Signaling Technology (Beverly, MA, USA). The glyceraldehyde phosphate dehydrogenase (GAPDH) antibody was obtained from Santa Cruz Biotechnology (Santa Cruz, CA, USA). The blots were developed using an enhanced chemiluminescence detection system (GE Healthcare, Piscataway, NJ, USA).

### MMP-9 promoter reporter assay

The MMP-9 promoter reporters, wild-type pMMP9-Luc(−925/+13), and disrupted NF-κB binding site pMMP9-Luc(−925/+13)mtNFκB are described elsewhere^31^. MDA-MB-231 cells cultured on 12-well plates were transfected with 0.5 μg of the MMP-9 promoter reporter constructs, along with 50 ng of the pRL-null plasmid encoding *Renilla* luciferase to monitor transfection efficiency. At 48 h post-transfection, the cells were treated with 10 ng/mL TNFα in the presence or absence of 10 μM DPP23. After 8 h, the cells were collected, and the firefly luciferase activities were measured as previously described^31^. Luminescence was measured using a Centro LB960 dual luminometer (Berthold Technologies).

### Immunofluorescence microscopy

MDA-MB-231 cells plated on coverslips were untreated or treated with 10 ng/mL TNFα in the presence or absence of 10 μM DPP23 for 30 min, followed by fixation, permeabilization, and incubation with primary antibodies against α-tubulin and phospho-p65 (Ser536) for 2 h, as previously described^8^. Secondary antibodies conjugated to Alexa Fluor 488 (Invitrogen; green signal for α-tubulin) and Alexa Fluor 555 (Invitrogen; red signal for phospho-p65) were incubated with the cells for 30 min, followed by staining of the nuclear DNA (1 μg/mL Hoechst 33258 (Sigma-Aldrich), blue signal) for 10 min. The stained cells were examined under an EVOS f1 fluorescence microscope (Advanced Microscopy Group; Bothell, WA, USA).

### Molecular docking simulations

Molecular docking experiments were performed on an Intel Core 2 Quad Q6600 (2.4 GHz) Linux PC with Sybyl 7.3 (Tripos, St. Louis, MO), as previously described^44^. 3D0E.pdb was used as the X-ray crystallographic structure of human Akt2^36^. The 3-D structure of DPP23 was determined using X-ray crystallography. All docking experiments were performed on an Intel Core 2 Quad Q6600 (2.4 GHz) Linux PC with Sybyl 7.3 (Tripos). The structure of the apo-protein of Akt2 (apo-3D0E) was prepared using the Sybyl program, and its solution structure was obtained by energy minimization using a previously reported detailed procedure^45^. The root-mean-square deviation between the crystal structure and the apo-protein was 0.1309 Å. The binding sites were determined based on the interpretation of the results analysed by the LigPlot program, as reported in the protein data bank^46^: Ala179, Lys181, Glu200, Met229, Ala232, Glu236, Asp293, and Phe294. A previously reported flexible docking method was used^45^. To confirm whether this docking process was correct, we docked 4-{2-(4-amino-1,2,5-oxadiazol-3-yl)-1-ethyl-7-[(3*S*)-piperidin-3-ylmethoxy]-*1H*-imidazo[4,5-c]pyridin-4-yl}-2-methylbut-3-yn-2-ol (GSK690693) as a reference ligand^36^. The radius for the active site was set to 1.6 Å. The docking process was iterated 30 times, and 30 complexes were generated (Supplementary Fig. S1a). Of them, 23 complexes were bound to the same site of the crystal structure. Their binding energy ranged from −21.94 to –10.42 kcal/mol. Similarly, DPP23 was docked into the apo-3D0E structure (Supplementary Fig. S1b). Thirty complexes bound well, and their binding energy ranged from −9.83 to –8.15 kcal/mol. 3-D images were generated using the PyMOL program (The PyMOL Molecular Graphics System, Version 1.3, Schrödinger, LLC).

### Lentiviral shRNA-mediated gene silencing

Lentiviral particles expressing a short hairpin RNA (shRNA) targeting Akt2 or a scrambled control were obtained from Sigma-Aldrich (MISSION^®^ shRNA; Sigma-Aldrich, St. Louis, MO, USA). MDA-MB-231 cells were transduced with lentiviral particles according to the manufacturer’s instructions. Knockdown of Akt2 protein expression was verified by immunoblot analysis.

### Syngenic experimental liver metastasis assay

Six-week-old female Balb/c mice were purchased from YoungBio (Seongnam, Gyeonggi-do, Republic of Korea). All animal experiments were conducted according to the standards and procedures approved by the Konkuk University Institutional Animal Care and Use Committee (no. KU15194). Balb/c mice were anaesthetized, and the spleen was exposed through a small incision in the left abdominal flank, as previously described^47^. The 4T1 mammary carcinoma cells (6 × 10^4^ cells/50 μL) were injected into the splenic lobe with a 30-gauge needle. The spleen was returned to the abdominal cavity, and the abdominal wall was closed using 6–0 nylon sutures. On day 1 post-implantation of the 4T1 cells, the mice were randomly divided into two groups. One group was intraperitoneally administered with PBS (n = 4), while the other group was intraperitoneally administered with 10 mg/kg DPP23 (n = 6) daily for 7 days. All mice were euthanized on day 8 after implantation, and the spleen and liver tissues were fixed with 10% phosphate buffered formalin and stained with haematoxylin and eosin (H&E).

### Statistical analysis

The data are presented as the mean ± S.D. Statistical analysis was analyzed by one-way analysis of variance (ANOVA) followed by Sidak’s multiple comparisons test using GraphPad Prism version 7.0 software (GraphPad Software Inc., La Jolla, CA, USA). *P* values less than 0.05 were considered statistically significant.

## Additional Information

**How to cite this article**: Shin, S. Y. *et al*. The UPR inducer DPP23 inhibits the metastatic potential of MDA-MB-231 human breast cancer cells by targeting the Akt–IKK–NF-κB–MMP-9 axis. *Sci. Rep*. **6**, 34134; doi: 10.1038/srep34134 (2016).

## Supplementary Material

Supplementary Information
