# Supplementary material for: The UPR inducer DPP23 inhibits the metastatic potential of MDA-MB-231 human breast cancer cells by targeting the Akt–IKK–NF-κB–MMP-9 axis
Source: Sci Rep. 2016 Sep 23;6:34134. doi: 10.1038/srep34134 (PMC5034246; doi:10.1038/srep34134)
Supplement: Supplementary Information [file srep34134-s1.pdf]

The UPR inducer DPP23 inhibits the metastatic potential of MDA-MB-231 human breast cancer cells by targeting the Akt–IKK–NF-κB–MMP-9 axis

Soon Young Shin[1,2], Chang Gun Kim[2], You Jung Jung[2], Yoongho Lim[3,*], and Young Han Lee[1,2,*]

[1]Department of Biological Sciences, College of Biological Science and Biotechnology, Konkuk University, Seoul 05029, Republic of Korea

[2]Cancer and Metabolism Institute, Konkuk University, Seoul 05029, Republic of Korea

[3]Division of Bioscience and Biotechnology, College of Biological Science and Biotechnology, BMIC, Konkuk University, Seoul 05029, Republic of Korea

Supplementary Figure S1

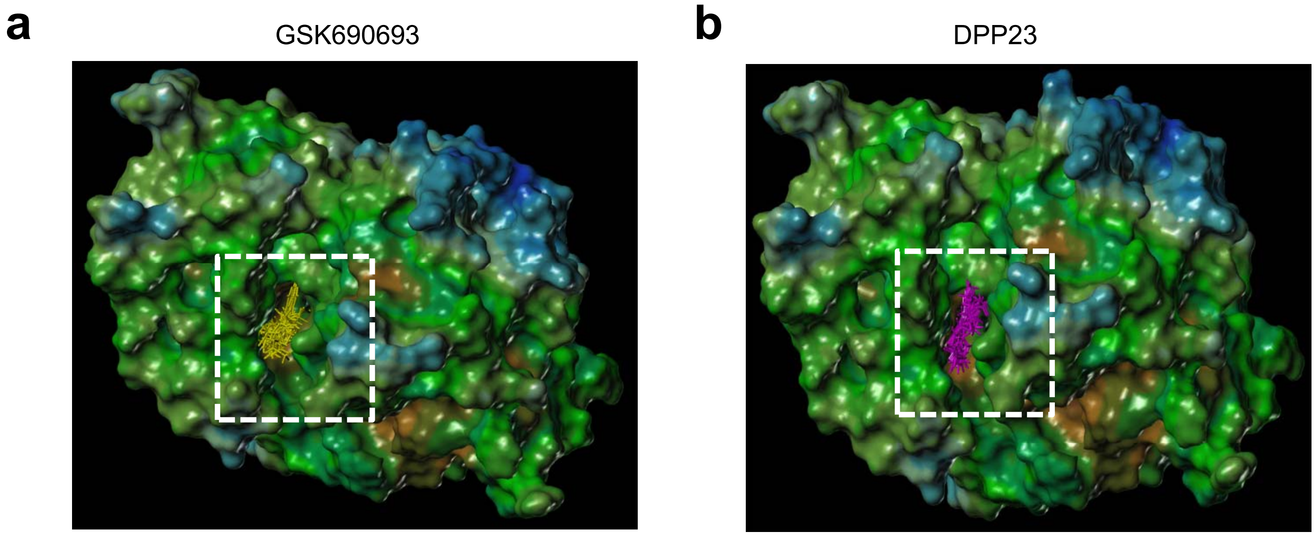

Supplementary Fig. S1. (a) Twenty-three GSK690693 molecules docked in apo-3D0E. (b) Thirty DPP23 molecules docked in apo-3D0E.
